# A review on research advances, issues, and perspectives of morels

**DOI:** 10.1080/21501203.2015.1016561

**Published:** 2015-03-09

**Authors:** Xi-Hui Du, Qi Zhao, Zhu L. Yang

**Affiliations:** Key Laboratory for Plant Diversity and Biogeography of East Asia, Kunming Institute of Botany, Chinese Academy of Sciences, Kunming, Yunnan650201, China

**Keywords:** *Morchella*, species diversity, species distribution, ecology, evolutionary history, cultivation, genome

## Abstract

Morels, a group of the world’s most prized edible and medicinal mushrooms, are of very important economic and scientific value. Here, we review recent research progress in the genus *Morchella*, and focus on its taxonomy, species diversity and distribution, ecological diversity, phylogeny and biogeography, artificial cultivation, and genome. We also discuss the potential issues remaining in the current research and suggest some future directions for study.

Due to its high economic and scientific value, the genus *Morchella* Dill. ex Pers.: Fr. has become a hot research topic in China and abroad in recent years. Based on the recent research achievements, we present a review of its taxonomy, phylogeny, species and ecological diversity, geographical distribution and biogeography, artificial cultivation, and genome.

## 1. Economic importance of morels

True morels (*Morchella* spp.) belong to the Pezizales, *Morchellaceae* (Hibbett et al. 2007), with *Morchella esculenta* (L.) Pers. as the type species. They are mostly distributed in temperate regions of the northern hemisphere where they typically fruit for only a few weeks each spring. Due to their desirable ﬂavor and short fruiting season, morels become the world’s most prized edible fungi. To meet the demand created by their growing popularity, wild morels, the main source of edible morels, are harvested commercially and exported extensively from China, India, Turkey, Mexico, and the United States (Pilz et al. 2007). In China, the annual export of dried morels increased ﬁvefold from 181,000 kg to 900,000 kg over the past 5 years, averaging $160 US dollars per kilogram.

## 2. Species diversity in *Morchella*

### 2.1. Morphological taxonomy of Morchella

According to the latest information in the Index Fungorum (http://www.indexfungorum.org/names/names.asp), 315 names in *Morchella* have been reported (including species, subspecies, and varieties). Most of them were described from Europe, with only few described in Asia and USA. Due to insufficient microscopic characteristics and high levels of variability in form and color of ascocarps during different developmental stages (Du et al. 2014), affected by ecological and climate factors, the species number in *Morchella* varies from 3 to 50 or more, which has caused confusing use of homonyms and synonyms (Bresinsky et al. 1972; Gessner et al. 1987; Volk and Leonard 1989; Jung et al. 1993; Bunyard et al. 1994, 1995; Kanwal et al. 2011; Clowez 2012; Kuo et al. 2012; Mortimer et al. 2012; Richard et al. 2014).

Based on gross morphology, the species of *Morchella* were initially placed into three groups: black morels, yellow morels and semi-free capped morels. Later, Guzmán and Tapia (1998) presented a fourth group, namely, blushing morels, including *M. rufobrunnea* Guzmán & F. Tapia *M. guatemalensis* Guzmán et al. and *M. rigidoides* R. Heim, distributed in the tropics or subtropics.

### 2.2. Molecular phylogenetic species of Morchella

Recent rapid developments in DNA-sequencing techniques and phylogenetic analysis have enabled mycologists to overcome difficulties in fungal taxonomy and systematics and elucidate the morphological, ecological, and functional evolution of fungi (Koufopanou et al. 1997; Geiser et al. 1998; Yang 2011, 2013). Development of Genealogical Concordance Phylogenetic Species Recognition (GCPSR) (Taylor et al. 2000) provided a consensus criterion for resolving species relationships among fungi, including those of morels (Dettman et al. 2003; Revankar and Sutton 2010; Taşkın et al. 2010, 2012; O’Donnell et al. 2011; Du et al. 2012a; Zeng et al. 2013; Elliott et al. 2014; Pildain et al. 2014; Voitk et al. 2014).

O’Donnell et al. (2011) first used the GCPSR method for conducting phylogenetic and biogeographic studies of the genus *Morchella* based on the LSU-*ef1-a-rpb1-rpb2* combined-gene dataset. This study presented the genus *Morchella* consisting of the Esculenta Clade (yellow morels), the Elata Clade (black morels) and the Rufobrunnea Clade (blushing morels), respectively, consisting of 16, 32, and 1 species. More importantly, their results indicated that semifree capped morels were deeply nested within the Elata clade (black morels), contradicting an earlier opinion that semifree capped morels were the separate genus *Mitrophora* (Breitenbach and Kränzlin 1984). Because morphological plasticity of morels complicates the application of Latin binomials with confidence to all these species, O’Donnell et al. proposed identifying species by clade (*Mel* for Elata, *Mes* for Esculenta) followed by a unique Arabic number for each species within the two clades. But in this study, samples from eastern Asia, especially from China, were underrepresented.

Based on a broad sampling of morels in China, Du et al. (2012a) further evaluated species diversity of *Morchella* and conducted a molecular phylogeny and biogeography study. Their analyses identified 61 species in the genus, including 27 species in the Esculenta Clade (*Mes-*1~*Mes-*27), 33 species in the Elata Clade (*Mel-*1~*Mel-*34, please note *Mel-*13 and *Mel-*26 have been combined into one species based on the analysis in this study), and one in the Rufobrunnea Clade. Their results also suggested that East Asia or China (ca. 30 species) is the center of diversity and distribution of the modern species of the genus (Du et al. 2012a).

Taşkın et al. (2012) reported *M. anatolica* described in Işiloğlu et al. (2010) also belonged to Rufobrunnea Clade which was represented by two extant species, *M. rufobrunnea* and *M. anatolica*.

Recently, three new species in the Elata Clade have been separately identified using ITS-*ef1-a-rpb1-rpb2* combined-gene sequences, namely, *Mel-*35 (*M. australiana* T.F. Elliott, Bougher, O’Donnell & Trappe) from Australia (Elliott et al. 2014), *Mel-*36 from Canada (Voitk et al. 2014), and *Mel-*37 from Argentina (Pildain et al. 2014).

Thus far, 65 phylogenetically distinct species have been recognized in *Morchella*.

### 2.3. Relationship between phylogenetic and morphological species of Morchella

According to recent studies, 29 phylogenetic species in Morchella could be given Latin binomials (O’Donnell et al. 2011; Kuo et al. 2012; Elliott et al. 2014; Richard et al. 2014). *Mes*-1, *Mel*-1, *Mel*-3, *Mel*-4, *Mel*-15, *Mel*-36, *M. anatolica*, and *M. rufobrunnea*, respectively, correspond to *M. steppicola* Zerova (Зерова 1941), *M. tomentosa* M. Kuo (Stefani et al. 2010), *M. semilibera* DC. (Fries 1822), *M. punctipes* Peck (Peck 1903), *M. angusticeps* Peck (Peck 1879), *M. australiana* (Elliott et al. 2014), *M. anatolica* (Işiloğlu et al. 2010), and *M. rufobrunnea* (Guzmán and Tapia 1998). Another 21 species have been described, respectively, in Kuo et al. (2012), Clowez (2012) and Rcihard et al. (2015, Figure 1). The remaining undescribed species need further intensive study on their morphological characters.

**Figure 1.**
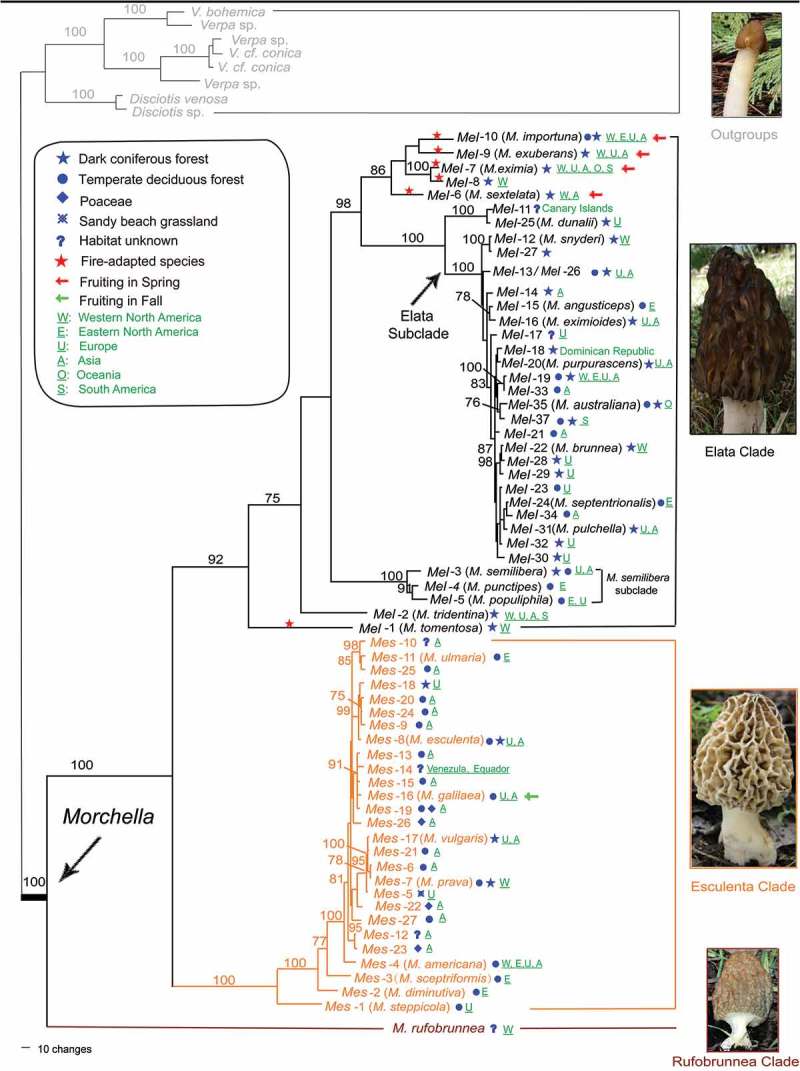
Phylogenetic tree* of *Morchella* inferred from a maximum parsimonious analysis of the combined ef1a-rpb1-rpb2 dataset. Verpa and Disciotis are used as outgroups. Bootstrap values (above 75%) are indicated above branches.

Note: *Sequences used here are, respectively, generated in Taşkın et al. (2010), O’Donnell et al. (2011), Du et al. (2012a), Elliott et al. (2014) and Pildain et al. (2014). *Mel*-36 and *M. anatolica* are not included on this tree due to their sequences unavailable in GenBank.

Note: Six species names described by Kuo et al. (2012) and Clowez (2012) have been discovered to be synonyms. Because the epithets proposed by Clowez (2012) have priority over those applied to conspecifics in Kuo et al. (2012), Richard et al. (2014) assess what taxa in the latter study represent nomenclatural synonyms of taxa validly published by Clowez (2012).

### 2.4. Dedicated web for rapid identification of Morchella phylogenetic species – Morchella MLST

Du et al. (2012b) observed that the ITS-*ef1-a-rpb1-rpb2* combined-gene data could successfully identify all the phylospecies in *Morchella*, with the ITS gene alone identifying 77.4% of them. Unfortunately, at least two-thirds of ITS sequences of *Morchella* in GenBank were found to be misidentified, a problem noted for other groups of fungi (Vilgalys 2003; Ryberg et al. 2008).

To minimize potential problems created by poorly annotated sequences in GenBank, and accurately identify known and novel species, the *Morchella* MLST database (multilocus sequence typing, http://www.cbs.knaw.nl/*Morchella*/) was constructed (Du et al. 2012b). Sequences generated in recent studies (Taşkın et al. 2010, 2012; O’Donnell et al. 2011; Du et al. 2012a, 2012b) have been recorded in this site. The scientific community not only can freely download these sequences and access information for voucher specimens and/or cultures from this website, but also can contribute their validated sequences and information to the site.

## 3. Species distribution and ecological diversity of *Morchella*

In this section, the results reported in Taşkın et al. (2010, 2012), O’Donnell et al. (2011), Du et al. (2012a, 2012b), Voitk et al. (2014), Elliott et al. (2014), Pildain et al. (2014), Beug and O’Donnell (2014) and Richard et al. (2014) have been integrated to provide a comprehensive picture of the species distribution and ecological diversity of *Morchella*.

### 3.1. Global distribution pattern of Morchella

Of the 65 phylospecies identified in *Morchella*, more than half are represented in East Asia or China (34 species) including 20 endemic species, most of them representing lineages that originated in the middle Miocene. Less species diversity was found in Europe than in East Asia. Of the 27 species present in Europe, 12 are endemic. Twenty-one species have been found in North America, but most of them were basal species, including 14 endemic species. Based on the latest published data, East Asia or China still served as the modern species diversity center of *Morchella*.

Among the 25% species with disjunct distributions in *Morchella, M. eximia* M. Kuo (*Mel-*7) is the most widely distributed species, found in western North America, Europe, Asia, Australia, and South America. The detailed distribution information of each species is shown in Figure 1.

Interestingly, high continental endemism and provincialism have been identified in species of the Esculenta Clade, nearly 60% of which were endemic in East Asia and only 11% of which had disjunct distribution. In contrast, broad distributions were detected in nearly 31% of the species of the Elata Clade, and a higher species diversity was found in Europe than in East Asia for this clade.

### 3.2. Distribution pattern of Morchella in China

Following Wu and Wu’s (1996) geographic divisions of Chinese seed plants, Du et al. (2014) divided the distribution of morels in China into seven regions. Of 30 species detected in China, 20 were distributed in the Sino-Japanese Forest Subkingdom, 17 in the Sino-Himalayan Forest Subkingdom, four in the Qinghai-Xizang Plateau Subkingdom, four in the Eurasia Forest Subkingdom and one in the Malesian Subkingdom. No representative was discovered based on the present survey in the Eurasia Steppe Subkingdom and the Central Asiatic Desert Subkingdom.

The Sino-Himalayan Forest Subkingdom and the Sino-Japanese Forest Subkingdom, as the two main morel-producing regions in China, harbor roughly equivalent numbers of species, but given that the area of the latter is three times that of the former, the Sino-Himalayan Forest Subkingdom was considered by Du et al. (2014) to have the highest species richness of morels and serve as the center of species diversity of this genus in China. We attributed this to its diverse habitats and environmental heterogeneity. But, we also found species of Esculenta Clade and species of Elata Clade, respectively, were mainly distributed in the Sino-Japanese Forest Subkingdom and in the Sino-Himalayan Forest Subkingdom.

### 3.3. Ecological diversity of Morchella

The trophic status of morels has for long been a source of scientific interest and debate. Buscot and Kottke (1990, 1993) and Dahlstrom et al. (2000) reported morels could form ectomycorrhizae with Pinaceae plants. Stark et al. (2009) speculated morels were associated with orchids, based on evidence obtained through PCR-amplification directly from root-extracted DNA and cloning of the PCR products. Hobbie et al. (2001) and Li et al. (2013) assessed the trophic status of morels by examining the relative abundance of stable isotopes. Hobbie et al. (2001) suggested that morels were largely saprophytic, but Li et al. (2013) suggested that morels with black pilei were saprophytic and those with yellow pilei were mycorrhizal. Baynes et al. (2012) detected *Morchella* as an endophyte in the aboveground stem tissue of cheatgrass and reported that *M. sextelata* M. Kuo and *M. snyderi* M. Kuo & Methven could infect cheatgrass roots. So far, the trophic strategies of *Morchella* have not been settled, but the available data seems to indicate that *Morchella* probably includes not only saprophytic species and mycorrhizal species, but also facultative mycorrhizal species.

Based on long-term field observations, the following ecological characteristics of *Morchella* have been noted.

#### 3.3.1. Phylogenetic niche conservation (PNC)

Both the Esculenta Clade and the Elata Clade have preferential habitats. In the Esculenta Clade, approximately 70% of the species are found within temperate deciduous forests, while a few species are found on sandy beach grassland or in proximity to bamboos or reeds. In the Elata Clade, approximately 70% of the species are found in coniferous forest, while a few species are found within temperate deciduous forests (Figure 1). When the two Clades began to diversify in the late Cretaceous (Du et al. 2012a), both temperate deciduous biome and coniferous biome were well established in the northern hemisphere (Axelrod 1960), so these niches have probably been conserved for the past 100 million years. Phylogenetic niche conservation (PNC, Donoghue 2008) in *Morchella* has been speculated to persist throughout its evolutionary history (Du et al. 2012a).

#### 3.3.2. Cold tolerance

The Elata Clade is preferentially distributed at higher altitudes (above 2000 m) whereas the Esculenta Clade is mainly distributed on lower mountains (below 1200 m) and plains (yet *Mes*-14 fruiting at high altitude in Venezuela). In addition, the preferred niches of the two clades were dark coniferous forests and temperate deciduous forests, respectively. Based on these habitat preferences, we suggest that species in the Elata Clade might have stronger cold tolerance than those in the Esculenta Clade. Svenning (2003) considered cold tolerant plants to be much more capable of thriving and becoming widespread during the severe Plio-Pleistocene extinctions in Europe. Likewise, the stronger cold tolerance of the Elata Clade might have contributed to its survival during the Quaternary Ice Age, which probably explains why there are more widespread species in the Elata Clade.

#### 3.3.3. Post-fire adapted ability

Several species within *Morchella* can fruit in post-fire habitats. These fire-adapted species proliferate mainly in coniferous forests following a wildfire during spring or summer, usually for 1 or 2 years, after which the yield rapidly declines and disappears. To date, four obligate fire-adapted species (*M. tomentosa, M. sextelata, M. eximia*, and *Mel-*8) collected on burned sites and two facultative fire-adapted species (*M. exuberans* M. Kuo & M.C. Carter and *M. importuna* M. Kuo, O’Donnell & T.J. Volk) collected on both burned and nonburned sites, were found in the Elata Clade (marked by red asterisks in Figure 1), while none in the Esculenta Clade. O’Donnell et al. (2011) and Du et al. (2012a) suggested these adaptive shifts evolved convergently and ecological speciation have likely played an important role during the evolutionary history of morels.

#### 3.3.4. Diverse fruiting seasons

Spring is the main fruiting season of *Morchella*, but a few species fruit in summer or fall. For example, in Yunnan, SW China, where spring-fruiting, summer-fruiting and fall-fruiting species are distributed, most species fruit in spring (from March to May), but three fire-adapted species in the Elata Clade fruit in summer (July, indicated by red arrows in Figure 1), and one species in the Esculenta Clade fruits in fall (October, indicated by a green arrow in Figure 1). Neven et al. (2014) suggested the variation in fruiting behavior (spring vs. autumn) in *M. esculenta* s. lat. might depend on differences in life cycle elements/phases, which could represent two different adaptive life strategies (ecotypes or species). Further in-depth studies are needed to elucidate the cause and mechanism of fruiting season diversity.

## 4. Evolutionary history of *Morchella*

The historical biogeography of *Morchella* was first studied by O’Donnell et al. (2011) whose work set a foundation for subsequent investigations. Further study by Du et al. (2012a) contributed to a comprehensive understanding of the evolutionary history of the genus, especially in identifying East Asia or China as the modern species diversity and distribution center. Additionally, Elliott et al. (2014) and Pildain et al. (2014) broadened the previous understanding of species diversity and distribution of morels in South America and Australia. These studies need to be integrated with other studies to systematically extend the biogeographic analysis. Here, we discuss the evolutionary history of *Morchella* mostly based on the work of O’Donnell et al. (2011) and Du et al. (2012a).

**Figure 2.**
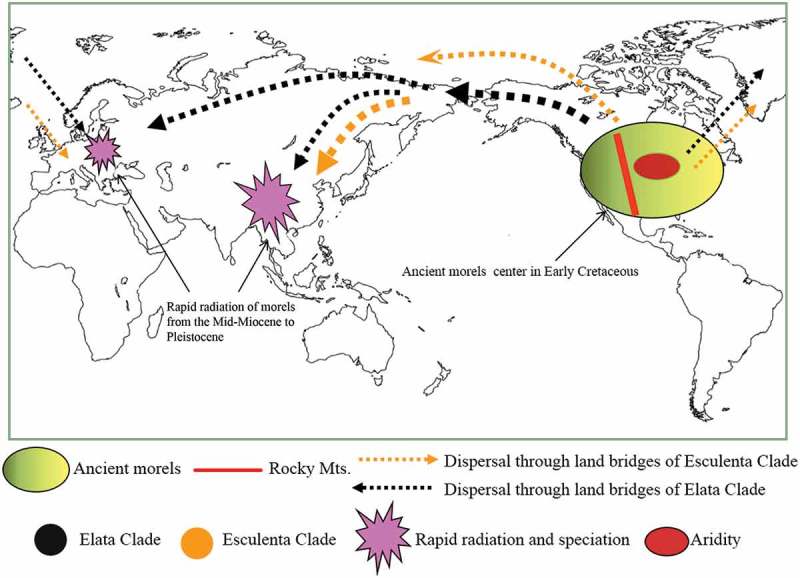
Hypothesis of place of origin, migration routes, and rapid radiation and speciation of morels.

It is speculated that *Morchella* originated in western North America (Figure 2) during the late Jurassic and diverged into the basal lineage *M. rufobrunnea*. In the early Cretaceous, the ancestors of the Esculenta and Elata Clades originated in western North America, and then spread to eastern North America. Due to the emergence of the Mid-Continental Seaway in the mid Cretaceous and the subsequent uplift of the Rocky Mountains during the late Cretaceous (Sanmartín et al. 2001), significant obstacles to gene flow among the morels formed between western and eastern North America, leading to the divergence of the ancestors of the Esculenta and Elata Clades diverging into the Elata Clade and the Esculenta Clade in western and eastern North America, respectively, during the late Cretaceous. New species evolved in eastern and western North America independently, with little exchange between the two regions (such as *M. americana* M. Kuo, Dewsbury, Moncalvo & S.L. Stephenson). Some basal species spread to Europe via the Thulean North Atlantic Land Bridge. Most species crossed the Beringian Land Bridge and dispersed to Asia. In the late Oligocene, and continuing in the Miocene, new folding of the Rocky Mountains and uplift of the Sierra Madre Oriental Range caused cooler and drier climates in central North America (Sanmartín et al. 2001; Donoghue and Smith 2004), which probably resulted in the widespread extinction of *Morchella* in that region.

During the middle Miocene to the Pleistocene, about 84% (51/61) of *Morchella* species rapidly evolved, including the radiational differentiation of 85% of the species of the Esculenta Clade in East Asia and 64% of the species of the Elata Clade in Europe. This differentiation might have been induced by the gradually cooling climate at mid- to high-latitudes of the North Hemisphere and the climate changes and environmental heterogeneity in East Asia caused by the rise of the Qinghai-Tibetan Plateau.

During the Quaternary glaciation, the abrupt cooling of the climate and the frequent fluctuations between the glacial and interglacial phases highly impacted the biological diversity and distribution. Refugia created by microclimatic variations provided some protection, and habitat fragmentation caused new speciation and diversification (Hewitt 2000; Harrison et al. 2001; Yang 2005). As indicated by the current published data, distribution areas of morels in North America and Europe are concentrated on the west and east coasts and the Mediterranean coast, which have been recognized as probable refugia in several studies (Hewitt 2000; Soltis et al. 2006). The Quaternary glaciation was not as severe and destructive in China as in Europe and some parts of North America, and complicated topography and more-or-less longitudinal arrangement of mountain ranges in China perhaps offered useful refugia for many ancient species (Yang 2005; Qiu et al. 2011), possibly explaining East Asia or China’s position as the center of species diversity and distribution of the modern *Morchella*.

## 5. Cultivation of morels

Morels are difficult to grow commercially. Five reports of their cultivation in the USA, Israel and China have been published (Ower 1982; Kuo 2008; Zhu 2008; Zhao et al. 2009; Masaphy 2010). The commercial cultivation of only *M. rufobrunnea* (Kuo 2008) and *M. importuna* (Zhao et al. 2009) has been successfully achieved separately in the USA and China. Whether other species of *Morchella* could be cultivated is unknown and deserves further research.

## 6. Genomics of *Morchella*

The worldwide demand for these delicious and highly prized edible mushrooms has stimulated intense efforts to cultivate morels. Sequencing the morel genome will provide unprecedented insights into its trophic status, sex, and fruiting at the molecular level. The identification of processes that condition and trigger fruit body formation will be uncovered by a thorough analysis of genomic traits, potentially leading to efficient commercial production. To date, the genome of only one species in the Elata Clade has been completed and reported in the 1000 Fungal Genomes project supported by the DOE Joint Genome Institute (http://genome.jgi.doe.gov/).

## 7. Issues and perspectives

Gratifying progress in the study of morels has been made in recent years, but there are still some problems which urgently need to be resolved. Currently, species delimitations of *Morchella* are defined using the criteria of GCPSR, while the morphological characteristics of many phylogenetic species without Latin binomials need deep and thorough investigation. We lack understanding of the speciation mechanisms in *Morchella* and the significance of ecological diversity. The key factors which permit the successful cultivation of *M. ruforbrunnea* and *M. importuna* need to be identified as well as whether other species in this genus could be cultivated.

Facing the challenges mentioned above, we could advance in the following directions: (1) more in-depth morphological studies need to be conducted to apply proper Latin binomials on unnamed phylogenetic species and popularize those names for effective scientific and commercial communication; (2) considering the diverse ecological habitats that support morels, it would be useful to select some species with unique habitats to do a trophic status study, moving morel research into an ecosystems perspective; (3) more genome studies should be on the agenda, comparing genetic mechanisms among species, especially with regard to mating type genes and reproductive modes, which will provide an effective theoretical and technical framework for the cultivation, protection and utilization of morels.

## Disclosure statement

No potential conflict of interest was reported by the authors.
